# Public Health Workforce Development^1^

**DOI:** 10.3201/eid1011.040797_09

**Published:** 2004-11

**Authors:** Rubina Imtiaz, Gail Cassell

**Affiliations:** * Centers for Disease Control and Prevention, Atlanta, Georgia, USA;; † Eli Lilly, Indianapolis, Indiana, USA

**Keywords:** public health workforce development, Uganda, health training, public health workers, Council of State and Territorial Epidemiologists

Until the early 1990s, most of Uganda's public health workforce obtained master's degrees from abroad. The responsibility for health training institutions has been shifting between the Ministry of Health and the Ministry of Education, although it is currently under the Ministry of Health, also the main employer of public health workers. The Ugandan Public Health School Without Walls is an innovative and sustainable model of worker development, conceived in 1994 in partnership with Makerere University, which houses the program; the Ministry of Health; and the development partners, notably, the Rockefeller Foundation, the Centers for Disease Control and Prevention (CDC), and the World Health Organization (WHO). Up to 145 professionals from medical, biologic, and social sciences have been trained in a 10-year period. The curriculum is flexible and is constantly reviewed and adapted to the local situation. The program is 75% field-based, during which trainees are placed in 15 district training sites under professional working conditions and they also rotate through various Ministry of Health programs, where they get hands-on training. The program emphasizes operational research, dissemination of findings to policymakers, and evidence-based management decisions, features that have translated into marked improvements in the quality of the delivery of health systems in the country. Students participate in both national and international outbreak investigations. Challenges include increasing the numbers of students to match the high demand and increasing the number of learning facilities. Ensuring effective mentorship, appropriate recruitment, career paths for graduates, and sustainability of the program with reduced donor funding are also problems.

Other examples of international public health worker development initiated by WHO and its Communicable Disease Surveillance Response unit for development of training materials in partnership with CDC are the Lyon 2-year training program for laboratory specialists and epidemiologists, Global Outbreak Alert and Response Network, and various internships.

The Council of State and Territorial Epidemiologists (CSTE) conducted an Epidemiology Capacity Assessment survey of all states and territories. As of November 2001, a total of 1,366 persons were employed as epidemiologists in the 44 responding state and territorial health departments; almost half (47.7%) of these epidemiologists were working in infectious disease. The survey found that 42% of all epidemiologists working in state and territorial health department have no formal academic training in that discipline. States reported that approximately 48% of epidemiologists work in infectious diseases (a figure that is close to optimal), but that the rest of the public health disciplines, such as chronic disease, maternal child health, occupational health, oral health, bioterrorism/emergency preparedness, injury, and environmental health, are far below optimal capacity; further, >60% of states epidemiologic funding support comes from federal sources. Most states reported having an insufficient number of epidemiology staff and resources to carry out essential public health services.

In response to the training needs identified by this assessment, CSTE, CDC, and Association of Schools of Public Health developed a 2-year applied epidemiology training fellowship that places trainees in state health departments. CSTE hosted the first national epidemiology workforce summit in January 2004 to identify strategies for building epidemiologic capacity in the U.S. public health system.

Infectious disease testing is one of the core capacities of public health laboratories. Such laboratories play a key role in supporting outbreak investigation and surveillance activities. Public health laboratory staff must meet unique requirements and posses technical skills that require a long learning curve. Staff also need to have the knowledge of public health principals and relevance of their work to public health activities. Special recruitment and retention issues are challenging the public health laboratory workforce, including increasing vacancy rates and an increasing demand for skilled workers in light of the Select Agent Rule. At the same time, technology is changing rapidly, with new tests emerging almost daily. Solutions offered were salary parity with the private sector, innovative training, creation of interest in laboratory sciences, and continuing education. The National Laboratory Training Network has helped by offering courses, and Emerging Infectious Diseases fellowships are also attracting new workers. In 2000, Association of Public Health Laboratories survey of state laboratory directors led to the "Green Book," which forecasts impending vacancies up to 40% in certain public health laboratory areas. This finding led to the development of the Center for Public Health Laboratory Leadership, which offers corrective courses and ventures.

